# Unusual Prostate-Specific Membrane Antigen (PSMA) Splenic Uptake in a Patient With Prostate Cancer

**DOI:** 10.7759/cureus.70751

**Published:** 2024-10-03

**Authors:** Hemanthkumar Athiraman, Katherine Miller, Jessica Guido, Yerko Borghero

**Affiliations:** 1 Hospital Medicine, Banner Health, Phoenix, USA; 2 Radiation Oncology, Banner MD Anderson Cancer Center, Gilbert, USA

**Keywords:** initial staging of prostate cancer, psma pet/ct, psma-pet imaging, prostate-specific membrane antigen, prostate cancer

## Abstract

There are many benefits to early detection of prostate cancer, including improved survival rates, less invasive treatment options, and better quality of life. Apart from traditional tests and imaging (including the prostate-specific antigen (PSA) test, digital rectal exam, biopsy, computed tomography (CT), and magnetic resonance imaging (MRI)), an imaging technique called prostate-specific membrane antigen positron emission tomography (PSMA PET) is more specific and sensitive, targeting PSMA expressed by prostate cancer cells on their surface.

This case report describes a 62-year-old male with metastatic prostate adenocarcinoma and unusual PSMA uptake in the spleen. Elevated PSA levels and MRI indicated aggressive prostate cancer, confirmed by a Gleason score of 7 from biopsies. A PSMA PET/CT scan showed intense activity in both the prostate and spleen, initially suggesting metastasis. However, further imaging identified the splenic lesion as a benign hemangioma. This case showcases the importance of thorough diagnostic evaluations for distinguishing metastatic disease from benign conditions to ensure accurate diagnosis and appropriate treatment.

## Introduction

Prostate cancer is the most common cancer in men, detected through elevated prostate-specific antigen (PSA) levels and confirmed by imaging and biopsy [1]. The staging and management of prostate cancer have been significantly improved by advanced imaging techniques such as magnetic resonance imaging (MRI) and prostate-specific membrane antigen (PSMA) positron emission tomography (PET)/computed tomography (CT). PSMA PET/CT offers high sensitivity and specificity for detecting both primary and metastatic disease [2]. However, it may yield atypical results, complicating the diagnostic process [3]. This case report highlights a 62-year-old male with prostate cancer whose rising PSA levels and PSMA PET/CT indicated potential splenic metastasis. Subsequent MRI suggested a benign hemangioma, underscoring the importance of thorough diagnostic evaluations in prostate cancer staging.

## Case presentation

**Figure 1:**
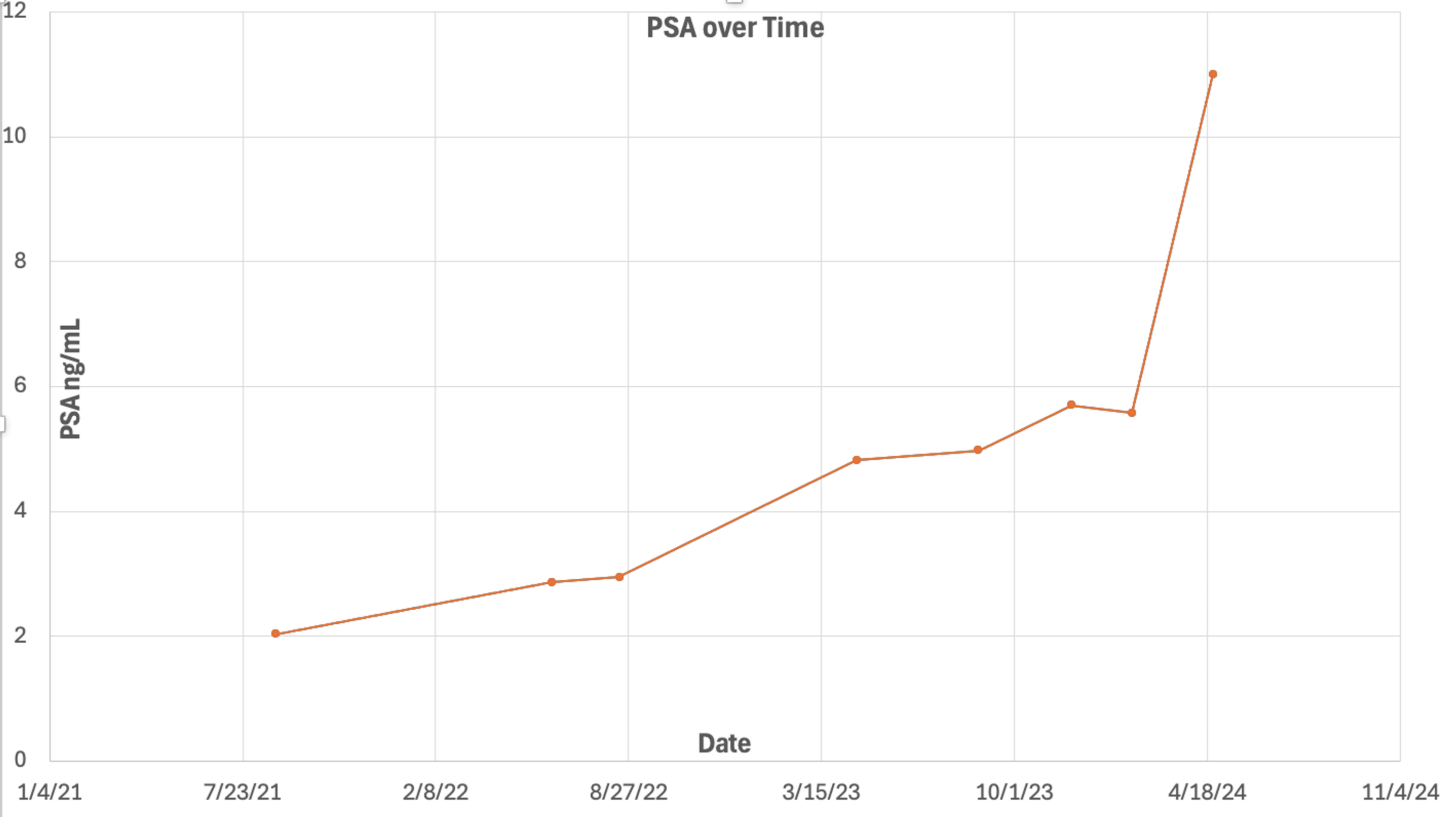
PSA levels over time. 8/26/21: 2.04 ng/mL; 6/8/22: 2.87 ng/mL; 8/17/22: 2.95 ng/dL; 4/21/23: 4.82 ng/dL; 8/25/23: 4.98 ng/dL; 11/29/23: 5.70 ng/dL; 1/31/24: 5.58 ng/dL; 4/24/24: 11.00 ng/dL. PSA = prostate-specific antigen

A 62-year-old male presented with progressively increasing PSA levels since July 2021 (Figure 1). He denied any history of prostatitis, prostate manipulation, or urinary tract infections. He had been on testosterone replacement therapy with pellets for testicular hypogonadism.

**Figure 2:**
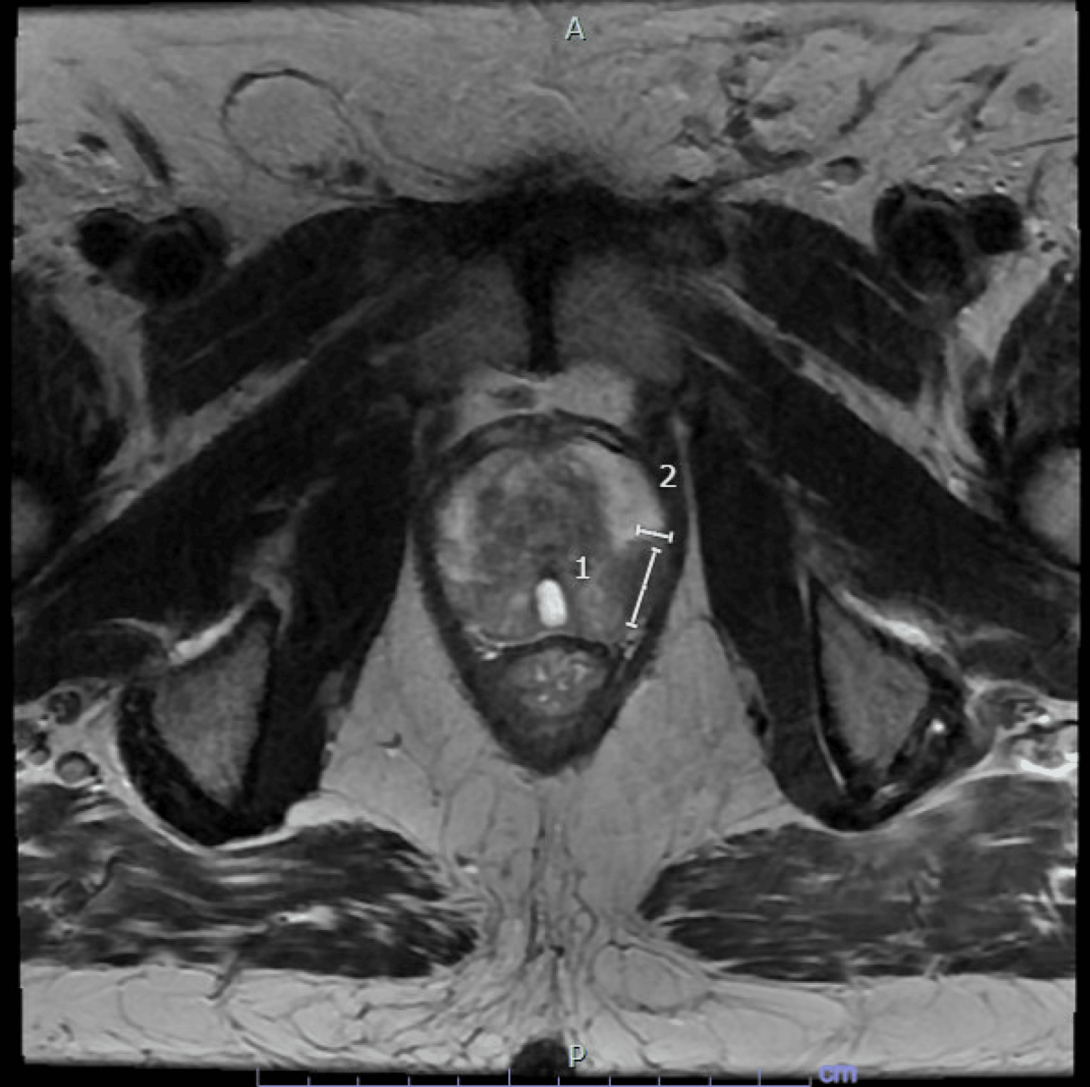
MRI of the prostate showed a lesion in the peripheral zone at the mid-gland (the length of the lesion is labeled as “1” and the width is labeled as “2”). MRI = magnetic resonance imaging

He underwent an extensive diagnostic workup. The 4Kscore is a blood test that measures four kallikrein protein levels (total PSA, free PSA, intact PSA, and human kallikrein 2) to assess the risk of aggressive prostate cancer [4]. In this case, the results of the 4Kscore test indicated a 21% probability of aggressive prostate cancer. The Prostate Imaging Reporting and Data System (PI-RADS) is a standardized reporting system for evaluating the prostate for cancer [5]. The MRI showed a PI-RADS 5 lesion in the left posterior lateral peripheral zone at the mid-gland (Figure 2).

The patient underwent cystoscopy, urethral dilation, and MRI fusion prostate biopsies. The pathology results were as follows: (1) left lateral base: adenocarcinoma, Gleason score 7, grade group 3 (40% involvement, area of interest 70%); (2) left lateral mid: adenocarcinoma, Gleason score 6, grade group 1 (80% involvement); and (3) left mid: adenocarcinoma, Gleason score 6, grade group 1 (25% involvement); left base: adenocarcinoma, Gleason score 6, grade group 1 (20% involvement). Eight additional cores showed benign prostate tissue.

**Figure 3:**
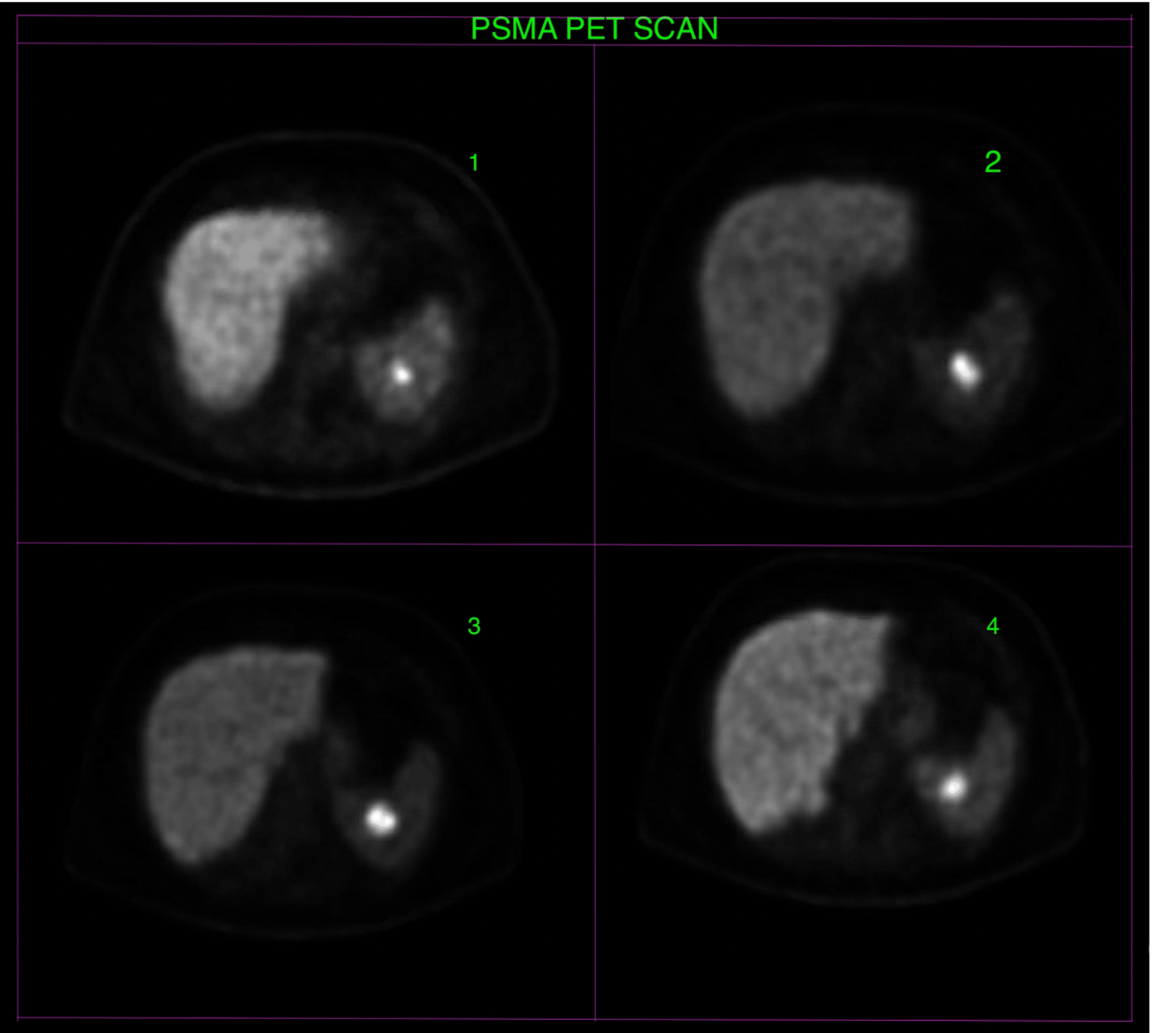
PSMA PET/CT images showing focal intense tracer activity in the spleen. PSMA = prostate-specific membrane antigen; PET/CT = positron emission tomography/computed tomography

**Figure 4:**
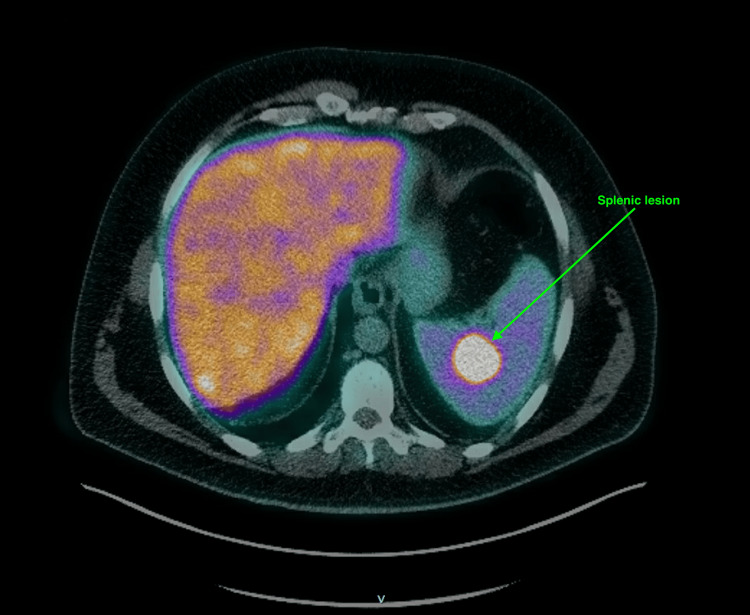
PSMA subtraction image showing radiotracer uptake in the spleen (green arrow). PSMA = prostate-specific membrane antigen

PSMA PET/CT scan demonstrated abnormal tracer activity in the posterior prostatic peripheral zone at the mid-gland and prostatic base, with the highest uptake intensity noted at the left posterolateral prostatic mid-gland peripheral zone. Additionally, there was focal intense tracer activity in the spleen consistent with metastatic prostate cancer based on tracer avidity and specificity for PSMA expression on prostate cancer cells (Figures 3, 4).

**Figure 5:**
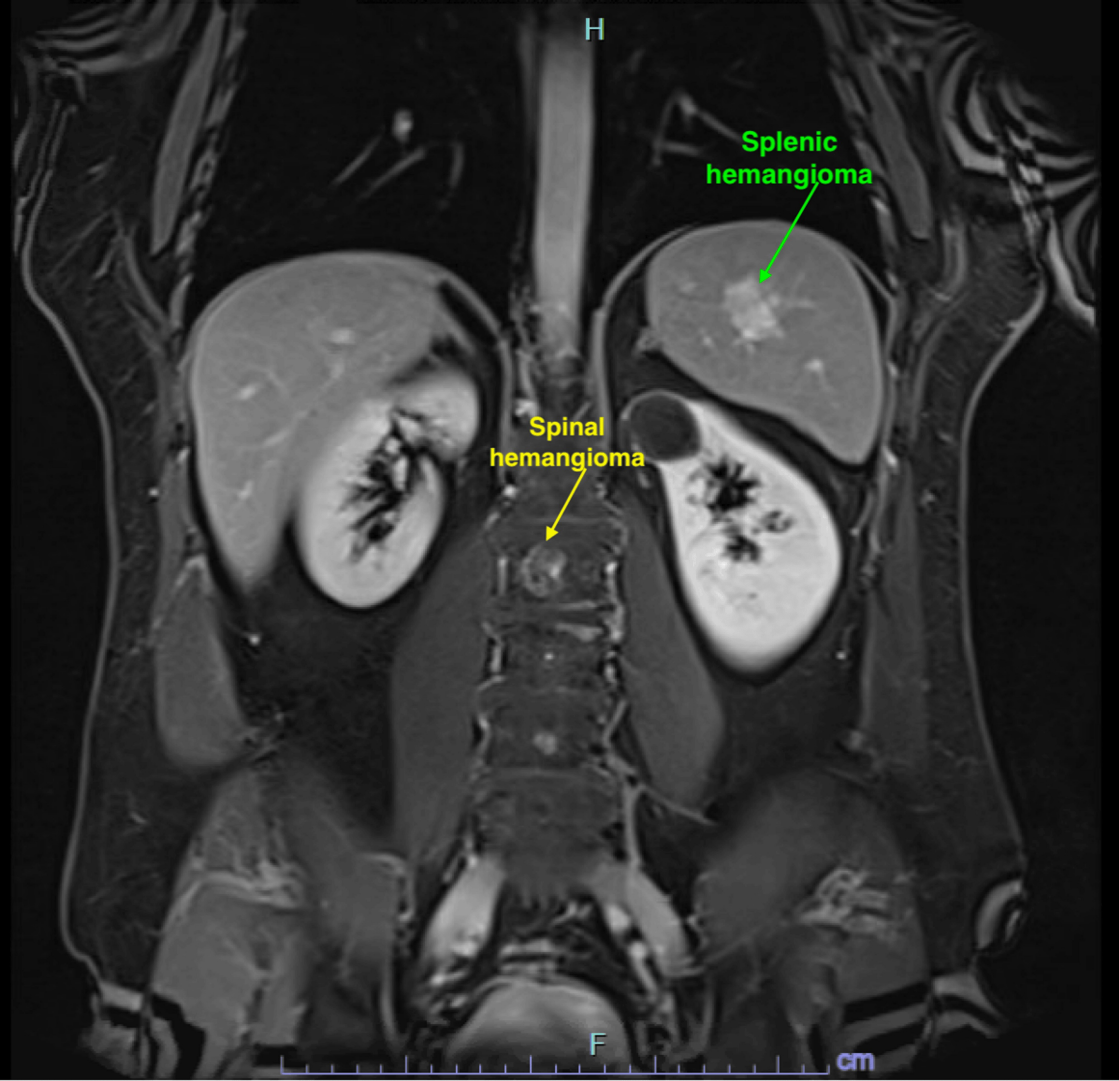
MRI of the abdomen showing splenic hemangioma (green arrow) and spinal hemangioma (yellow arrow). MRI = magnetic resonance imaging

To further evaluate the suspicious splenic lesion identified on the PSMA PET/CT, an MRI of the abdomen was performed. The MRI findings indicated that the splenic lesion exhibited imaging characteristics consistent with a hemangioma rather than metastatic disease (Figure 5). Additionally, lumbar spine hemangiomas were noted.

## Discussion

This case underscores the complexity of diagnosing and staging prostate cancer, particularly with atypical imaging findings. The patient’s rising PSA levels and subsequent biopsy confirmed the presence of prostate adenocarcinoma, Gleason score 7. The unexpected PSMA PET/CT findings of focal uptake in the spleen initially suggested possible metastasis, which was later clarified as a benign hemangioma through comprehensive abdominal MRI. Splenic hemangiomas are the most common benign spleen tumors [6]. 

The patient’s history of hypogonadism and use of testosterone replacement therapy were noted but did not appear to influence the imaging findings. It is important to note that a team-based approach with urologists, radiation oncologists, radiologists, medical oncologists, and pathologists, along with multiple imaging modalities including PSMA PET/CT and MRI, and biopsy plus laboratory data, are required to make a proper diagnosis and stage prostate cancer.

Published case reports show PSMA uptake in inflammatory and infectious diseases such as neurocysticercosis, tuberculosis, sarcoidosis, osteomyelitis, and nodular fasciitis; benign tumors such as meningioma, schwannoma, neurofibromas, thyroid and parathyroid adenomas, angiolipoma, hemangiomas, thymomas, pseudoangiomatous stromal hyperplasia of the breast, adrenal adenoma, pancreatic serous cystadenoma, intramuscular myxoma, desmoid tumor, and acrochordon; and malignant tumors such as gliomas, thyroid cancer, breast cancer, lung cancer, hepatocellular cancer, pancreatic cancer, neuroendocrine cancer, gastric adenocarcinoma, endometrium cancer, Ewing sarcoma, osteosarcoma, and multiple myeloma, among many others [7].

Important to note is an Australian study by Dr. Hofman and his team published in *The Lancet*, which showed PSMA PET/CT technique had 92% accuracy at detecting metastases versus 65% accuracy with traditional CT and bone scans [8]. Furthermore, the radiation exposure from PSMA PET/CT was substantially lower than from CT and bone scans [8]. Clearly, PSMA PET/CT is a powerful and valuable imaging tool. However, it can show malignant and benign conditions.

## Conclusions

PSMA PET/CT is a valuable tool in the staging of prostate cancer as it has sensitivity and specificity for detecting both primary and metastatic disease. However, this case highlights the need for a multidisciplinary approach, as well as comprehensive testing, including further imaging such as MRI and PSA level, to confirm the staging of prostate cancer. The PSMA avidity in the spleen initially thought to be metastasis was later identified to be a hemangioma via MRI. This misdiagnosis was avoided through comprehensive testing, which integrated multiple imaging techniques, along with blood tests and clinical pictures. It is important to note benign reasons for PSMA avidity while utilizing PSMA PET to stage prostate cancer.
